# Prevalence of Antenatal Depression and Associated Factors among Pregnant Women Attending Antenatal Care at Health Institutions of Faafan Zone, Somali Region, Eastern Ethiopia

**DOI:** 10.1155/2021/2523789

**Published:** 2021-08-27

**Authors:** Edao Tesa Keliyo, Meka Kedir Jibril, Girma Tadesse Wodajo

**Affiliations:** ^1^Department of Nursing College of Medicine and Health Science, Jigjiga University, Jigjiga, Ethiopia; ^2^Department of Public Health College of Medicine and Health Science, Jigjiga University, Jigjiga, Ethiopia

## Abstract

**Background:**

Depression is a common global mental health tragedy which affects more than 30 million people of all ages. Antenatal depression is higher among low-income countries where maternal and psychosocial factors act as determinant factors for its occurrence.

**Aim:**

This study is aimed at assessing the prevalence of antenatal depression and its associated factors among pregnant women attending health institutions of Faafan zone of Somali regional state, Eastern Ethiopia.

**Method:**

An institutional-based cross-sectional study design was conducted among randomly selected 403 pregnant women from January to September 2015. EPDS with 13 cutoff points was used to screen antenatal depression. Bivariate and multivariate logistic regressions were used to identify associated factors.

**Result:**

The study showed that 24.3% of women had antenatal depression. Marital status, educational status, chronic medical illness, previous depression history, and social support were factors associated with antenatal depression.

**Conclusion:**

The study revealed that the prevalence of antenatal depression was 24.3%. Ethiopia Federal Ministry of Health and Somali Regional Health Bureau should work very hard to create awareness on the importance of pregnancy planning and social support during pregnancy.

## 1. Introduction

Depression is a common global mental health tragedy, which can manifest with depressed mood, feeling of guilt, loss of interest, lack of sleep, and low self-esteem. Globally, it affects more than 30 million people of all ages [1]. By 2030, depression is predicted to be the second leading cause of morbidity in developing countries. A systematic review study indicated that antenatal depression is higher among low-income countries along with maternal and psychosocial factors as determinant factors for its occurrence [2, 3]. Prenatal depression encompasses major and minor depressive episodes beginning during pregnancy which can be characterized by antenatal depression symptoms like depressed mood, anxiety, poor fetal attachment, loss of concentration, and feelings of low self-esteem [4, 5]. Depression is the leading disease for women between the ages of 15 and 44 years in both high- and low- and middle-income countries [6]. Depression is currently the leading cause of nonfatal burden when considering all mental and physical illnesses, accounting for approximately 10% of total years lived with disability in low- and middle-income countries. By 2030, depression alone is likely to be the third leading cause of disease burden in low-income countries (4.7%) [7, 8]. Some women may experience their first depressive episode during pregnancy, whereas others with a history of depression are at increased risk for its recurrence, continuation, or exacerbation [9, 10].

Depression in pregnancy is associated with adverse child outcomes and places women at greater risk for inadequate prenatal care, alcohol use, and poorer weight gain in pregnancy: each of these factors affects the unborn infant and slows fetal growth. The effects can also continue until adolescent time [11–13]. Most studies of maternal depression have focused on postnatal depression. However, depression is the most prevalent psychiatric disorder during pregnancy [14]. According to a recent systematic review of cohort studies, the prevalence of antenatal depression was 14% compared to a 10.5% pooled prevalence of postnatal depression. Most antenatal depression prevalence rates found in studies in developing countries were around 20% [15, 16]. In Ethiopia, more than one in ten pregnant women and one in 20 postnatal women suffer from undetected depression and around half of them have thoughts of ending their life [17].

In Ethiopia, perinatal mental disorders contribute to maternal morbidity, in terms of poorer health, increased disability, and prolonged labor [18].

A study conducted in Turkey reported that depressive symptoms were found in 27 (21.4%), for the women with no formal education. Women who were dissatisfied with their marriage (38, 14.5%) were more likely to be depressed than women who were satisfied (33, 8.5%). It was found that BDI scores were >17 in women who had no perceived support from a significant person (12, 24.0%). Overall, 24 (24.2%) women with unplanned pregnancy had a significantly higher rate of depression than the planned pregnancy groups (47, 8.5%). The regression analysis showed factors that affect AD as follows: lower education level of women, unplanned pregnancy, and lack of social support during pregnancy [19].

A cross-sectional study done in Tanzania shows that having a *previous depressive episode* (OR 4.35, *P* < 0.01), low (OR 2.18, *P* < 0.01) or moderate (OR 1.86, *P* = 0.04) satisfaction with ability to access basic needs, conflicts with the current partner (OR 1.89, *P* < 0.01), or booking earlier for ANC (OR 1.87, *P* = 0.02) were independent predictors of antenatal depression in the logistic regression model [20]. According to the study done by using EPDS (>13 cutoff point) in Addis Ababa, Ethiopia, antenatal depression was significantly higher among women who had not planned their current pregnancy. Those women who had not planned their current pregnancy were 2.78 times more likely to have antenatal depression than those who had planned their pregnancy (AOR = 2.78 (95% CI: 1.59–4.85)). Absence of support from the baby's father was also associated with higher odds of having antenatal depression. Pregnant women who experienced lack of support from the baby's father had 89% higher odds of having antenatal depression when compared with women who got support from the baby's father (AOR = 1.89 (95% CI: 1.06–3.36)).

History of depression was found to be one of the factors associated with antenatal depression. Those pregnant women who had previous history of depression were nearly three times at higher odds of having depression as compared to pregnant women who had no history of depression (AOR = 2.57 (95% CI: 1.48–4.48)). Educational level, community's support, and partner's feeling on the current pregnancy were not significantly associated with depression during pregnancy in the multivariable logistic regression model [21].

The few prevalence studies among pregnant women in SSA suggest that the probable or definite psychiatric morbidity is high and also in Ethiopia. Perinatal mental disorders contribute to maternal morbidity. There is evidence that a number of risk factors are associated with maternal depression. Risk factors include the following: substance use, previous history of depression, age, poor marital relationships, low social status, poor social support, and unplanned or unwanted pregnancy [19, 22].

So far, few studies have been conducted to assess antenatal depression and associated factors in Ethiopia, but a study conducted in pastoralist community like Somali regional state and specifically Faafan zone is lacking. As Somali region has limited health service, a study which addresses the topic is much needed. This study attempted to examine the prevalence and factors associated with antenatal depression in Faafan zone, Somali regional state, Ethiopia.

The objective of this study was to assess the prevalence of depression and its associated factors among pregnant women attending ANC at health institutions of Faafan zone of Somali region, Eastern Ethiopia.

## 2. Methods and Materials

### 2.1. Study Design and Population

The study was conducted in Faafan zone of Ethiopian Somali region, Eastern Ethiopia, from January to September 2015. Ethiopian Somali regional state is located in the southeast part of the country. Somali region has nine administrative zones, namely, Faafan, Siti, Fik, Dhagahbur, Korrahe, Warder, Gode, Afder, and Liban. Faafan zone has 5 hospitals and 12 health centers. Maternal health services are free of charge in Ethiopia. ANC is provided in all selected health facilities [23]. ANC from a skilled provider is important to monitor pregnancy and reduce morbidity and mortality risks for the mother and child during pregnancy, delivery, and the postnatal period. According to Ethiopian Demographic and Health Survey (EDHS) of 2011, 34 percent of women who gave birth in the five years preceding the survey received ANC from a trained health professional at least once for their last birth. ANC utilization in Somali region was 21 percent. Institutional-based cross-sectional study design was employed among 403 pregnant mothers attending at health institutions in Faafan zone. The study populations were selected pregnant women attending ANC at the selected health institutions of Faafan zone from January to September 2015.

### 2.2. Sample Size and Sampling Procedures

The sample size was calculated using the single population proportion formula with the assumption: proportion of antenatal depression to be 50% since we could not find previous research with the same setting in Ethiopia, 95% certainty, 5% for *d*, and 10% for nonresponse rate. Accordingly, the calculated sample size became 403.

Institutions were stratified based on their service capacity into hospital and health centers. One hospital and seven health centers were selected randomly from all health facilities found in Faafan zone. The proportional sample size was allocated to selected health institutions based on their average daily ANC case flows. All consented ANC attendant women were taken into the study until the sample size was reached. Any apparently healthy pregnant women at any age of gestation who had come to the selected health institutions during the study period were included in the study. However, pregnant women who were critically ill, laboring, and unable to hear or speak during data collection were excluded from the study.

### 2.3. Data Collection

#### 2.3.1. Instruments

The Geriatric Depression Scale (GDS) is an evaluation tool in the diagnosis of depression. It is validated with Cronbach's alpha 0.84, test-retest reliability 0.91, and the concurrent validity of 0.83 to diagnose older adult's depression [24]. It is also validated with internal alpha 0.903 and test-retest reliability (ICC = 0.941 (95% CI: 0.886–0.970)) in Parkinson's disease [25]. EPDS has been also used to detect depressive symptoms [26]. The EPDS is a 10-item questionnaire, scored from 0 up to 3 (higher score indicating more depressive symptoms), that has been validated for detecting depression in antepartum and postpartum in many countries. The instrument was also validated in public health facilities in Ethiopia for postpartum use and showed sensitivity of 84.6% and specificity of 77.0% at the cutoff score 7/8 [22]. The cutoff point of EDPS among pregnant women is usually higher than postpartum women [27]. In Ethiopia, the EPDS cutoff point of 13 is used to identify pregnant women with depressive symptom [21]. We use EPDS for the purpose of this study.

Pregnant women who scored 13 and above were considered depressed women while pregnant women who scored below 13 were classified as nondepressed women [21]. Social support was measured using the Oslo-3 Social Support Scale (OSS-3) with three questions.

The response categories were assessed independently for each of the three questions, and a sum score was created by summarizing the raw scores.

The Oslo-3 scale has been used in several studies, thus confirming its feasibility and predictive validity with respect to psychological distress. In this study, the scale is used as both a sum score and an item-by-item scale.

The sum score scale ranging from 3 to 14 was used, which was then operationalized into three broad categories: “poor support” (3–8), “moderate support” (9–11), and “strong support” (12–14). A structured questionnaire was used to collect sociodemographic characteristics and clinical factors. Baseline clinical information was traced from the client's record by using checklists/data extraction formats.

### 2.4. Data Collection Tools and Procedures

The Somali version of adopted Edinburgh Postnatal Depression Scale which has ten items was used to collect data. A questionnaire adopted in English was translated to local Somali language by a language expert and then translated back to English to check consistency and accuracy. The Somali version of the questionnaire was used to collect data. Before conducting full study, a pretest was conducted among 21 clients (5% of the sample size) attending in Lefaisa health center. All necessary modifications were made before the actual data collection. Data was collected by trained, experienced eight BSc nurses under the supportive supervision of principal investigators and two trained supervisors. A three-day training was given to the data collectors and supervisors on the data collection tool, interview technique, eligible study subjects, and consent. History of chronic illness was taken from medical records of the clients.

### 2.5. Data Processing and Analysis

First, the collected data was checked for completeness and consistency. Then, it was coded and entered in to Epi Info version 3.5.3. Then, data was exported and analyzed by using SPSS version 16. Descriptive statistic was used to determine the prevalence of antenatal depression.

Binary logistic regressions were conducted between the predictors and antenatal depression. Using significant variables (*P* < 0.05) from binary logistic regression models, a multivariable logistic regression model was fitted to identify the independent predictors of antenatal depression. The strength of association was measured by odds ratios with 95% confidence intervals. All tests were two sided, and statistical significance was declared at *P* < 0.05.

### 2.6. Operational Definition

- *Depression*. Women having pregnancy scores 13 out of 30 points were considered having antenatal depression
- *Clinical Factors*. It is defined as gestational age, parity, maternal depression from previous pregnancy, previous history of depression and family history of depression, complications during current pregnancy, medication during pregnancy, previous poor pregnancy outcomes, and chronic medical illness
- *Substance Use*. It is defined as current use when patients use a specified substance in the last 12 months
- *Psychosocial Factors*. Social support is defined as “poor support” (a score of 3–8), “moderate support” (a score of 9–11), and “strong support” (a score of 12–14)
- *Chronic Medical Illness*. It is defined as cases such as diabetic mellitus, hypertension, gynecological disorders, lung disorders, and urinary tract infection

### 2.7. Ethical Consideration

Ethical clearance was obtained from the directorate of research and community service of Jigjiga University. Permission letter was taken from Somali Regional Health Bureau. Support letters were obtained from selected health institutions. Prior to data collection, written informed consent was obtained from the participants after a detailed explanation of the study and the study procedures. Only participants who gave written consent were interviewed in a separate room. Personal identifying details were not recorded. Participants identified with depressive symptoms were advised to visit the psychiatric clinic for better evaluation and treatment.

## 3. Results

### 3.1. Sociodemographic Characteristics

Three hundred ninety-five respondents participated in this study making the response rate of 98%. The mean age of study participants was 25.71 years (SD ± 5.04) with the age range of 15 to 40 years. Three hundred twenty-one (81.3%) participants were married and living together with their husband. Two hundred eight (52.7%) of study participants were illiterate, and more than eighty percent of study subjects were urban residents (Table 1).

### 3.2. Obstetric and Clinical Characteristics

Most of the study participants (70.9%) were in the second and third trimester at the time of the study. One hundred nine (27.6%) study participants had chronic medical illness. One hundred fifty-nine (39.5%) participants were taking medication during the study period, and 29 (7.3%) participants had developed complication during current pregnancy. One-fifth of the pregnant women (84, 21.3%) had experienced previous poor pregnancy outcome. Majority (357, 90.4%) of the respondents reported that their current pregnancy was planned/wanted (Table 2).

### 3.3. Psychiatric History, Social Support, and Substance Use

The study revealed that 41 (10.4%) of the study participants had a previous history of depression, and 19 (4.8%) of the study participants reported presence of history of depression in their family. Fifty-one (12.9%) participants had poor social support. Among the respondents, 44 (11.1%) had used Khat while 17 (4.3%) had used tobacco product at least once in the last twelve months. The percent of pregnant women who had used alcohol and cannabis at least one time in the last twelve months during the study was 2 (0.5%) and 1 (0.3%), respectively (Table 3).

### 3.4. Prevalence of Antenatal Depression

The internal consistency of the EPDS tool was acceptable (Cronbach′s *α* = 0.77). The study revealed that the overall prevalence of antenatal depression in this study was 24.3%.

### 3.5. Factors Associated with Antenatal Depression

Factors associated with antenatal depression were identified by using logistic regression. During bivariate logistic regression, variables like residence, monthly income, marital status, education, current pregnancy complication, chronic medical illness, number of pregnancy, pregnancy status, family history of depression, previous history of depression, previous history of antenatal depression, social support, and Khat use were associated with antenatal depression. However, factors like age, occupation, duration of pregnancy, previous poor pregnancy outcome, alcohol use, tobacco use, and cannabis use were not associated with antenatal depression. Multilogistic regression revealed that mothers who were married but not living together with their partners were three times more likely to get antenatal depression than those mothers who are married and living together with their husbands (AOR 2.9 1 (95% CI: 1.36, 6.23)).

Those women who had chronic medical illness were two times more likely to have antenatal depression than those who had no chronic depression (AOR = 2.21 (95% CI: 1.09–4.45)). Absence of social support was also associated with higher odds of having antenatal depression when compared with women who had social support (AOR = 3.34 (95% CI: 1.50–7.43)). Pregnant mothers who had previous history of depression were six times at higher odds of having antenatal depression as compared to pregnant women who had no history of depression (AOR = 6.02 (95% CI: 2.31–10.01)). Educational level, number of pregnancy, and previous history of depression were also factors associated with antenatal depression (Table 4).

## 4. Discussion

This study identified sociodemographic factors, obstetric factors, clinical factors, social support factor, and substance use factors that were associated with antenatal depression. The study revealed that the overall prevalence of antenatal depression was 24.3%. Marital status, educational status, chronic medical illness, number of pregnancy, previous depression history, and social support were factors associated with antenatal depression.

The level of antenatal depression found in this study is relatively lower as compared to findings of studies done in Addis Ababa, Ethiopia, that revealed 24.94% prevalence of antennal depression [28]. Even though there is no tangible evidence for this difference, the difference in the sample size of studies and sociodemographic characteristics may contribute for this variation. Similarly, the prevalence of antenatal depression in this study is lower than the finding of the study conducted in other parts of Africa such as Cape Town and Tanzania which reported the prevalence of 39% and 39.5%, respectively [20, 29]. However, the finding of this study is higher than the finding of other studies done in different parts of the world such as 4.4% in China [30] and 18.3% in Bangladesh [31]. These variations may be attributed to differences in sociodemographic and obstetric factors. Different studies conducted in various parts of the globe revealed that the impact of factors such as educational status and pregnancy planning affects the level of antenatal depression. The difference in antenatal depression in this study and study conducted in Addis Ababa may be attributed to the difference in the contraceptive prevalence rate. For instance, the contraceptive prevalence rate in Addis Ababa town (62.5%) is higher than that of Somali region (4.1%) [27].

Studies have shown that chronic medical illness and previous history of antenatal depression factors are associated with antenatal depression in Ethiopia and other parts of the world [32]. Similarly, this study revealed that the odds of antenatal depression were two times among the mother who had chronic medical illness during the study. This is similar with the studies conducted in Durban, Rio de Janeiro, Brazil [28, 33]. Previous history of depression was the largest antenatal depression contributor in this study.

In this study, previous history of depression was significantly associated with antenatal depression. This finding is similar with a study conducted in Addis Ababa which reported a significant association between previous history of depression and antenatal depression [32].

This study indicated that social support is also another factor associated with antenatal depression. The finding of this study is similar with the study conducted in Addis Ababa which showed that the odd of developing antenatal depression was 89% higher in those pregnant women who experienced lack of baby's father support [32]. The possible reason may be women who have social support during pregnancy may have low stress compared to women with poor social support. This study also found that the odds of antenatal depression among mothers who cannot read and write were two times higher than mothers who have some level of education. This is similar with other studies conducted in Turkey which showed that lower education level of women was a factor that affects antenatal depression [19]. This may due to the reason that educated mothers have higher access to reproductive health information, and this in turn may reduce stress during pregnancy.

## 5. Limitation of the Study

The study did not comprehensively address the possible factors associated with antenatal depression. The direct translation of EPDS was used but the psychometric properties of the tools were not validated in Ethiopia. Data for chronic medical illness was taken from records so that there is a possibility to make bias due to the nature of secondary data.

## 6. Conclusion and Recommendations

The study has assessed the prevalence of antenatal depression and associated factors. The study revealed that one-fifth of study population had antenatal depression. Marital status, educational status, chronic medical illness, number of pregnancy, previous depression history, and social support were factors associated with antenatal depression.

Ethiopia Federal Ministry of Health and Somali Regional Health Bureau should work very hard to create awareness about the importance of contraceptive use in the study area. Health education should be given to the community on the importance of pregnancy planning and social support during pregnancy at all levels. Ethiopian Ministry of Health and Somali Regional Education Bureau should work cooperatively to boost women's educational status. ANC providers at all levels should provide counseling, medical care, and emotional support to pregnant women.

## Figures and Tables

**Table 1:** Distribution of pregnant women by sociodemographic factors attending ANC at health institutions of Faafan zone, December 2015 (*n* = 395).

Variables	Categories	Frequencies	Percent (%)
Age	15-24	150	38.0
	25-34	218	55.2
	35-44	27	6.8
	Total	395	100.0

Residence	Urban	330	83.5
	Rural	65	16.5
	Total	395	100.0

Religion	Muslim	351	88.9
	Orthodox	32	8.1
	Protestant	12	3.0

Marital status	Married and living together	321	81.3
	Married but not living together	50	12.7
	Single	6	1.5
	Divorced	14	3.5
	Widowed	4	1.0

Ethnicity	Somali	272	68.9
	Oromo	62	15.7
	Amhara	36	9.1
	Gurage/Tigre	25	6.3

Education	Illiterate	208	52.7
	Primary education	112	28.3
	Secondary education and above	75	19.0

Occupation	Housewife	219	55.4
	Merchant	54	13.7
	Farmer	41	10.4
	Employee	67	17.0
	Student	14	3.5

**Table 2:** Obstetric and clinical characteristics of pregnant women attending ANC at health institutions of Faafan zone, December 2015 (*n* = 395).

Variables	Categories	Frequencies	Percent (%)
Chronic medical illness	Yes	109	27.6
	No	286	72.4
	Total	395	100.0

Number of pregnancy	First	77	19.5
	Second	120	30.4
	Third	90	22.8
	Fourth	32	8.1
	Fifth and above	76	19.2
	Total	395	100.0

Pregnancy status	Planned and wanted	357	90.4
	Unplanned or unwanted	38	9.6
	Total	395	100.0

Previous poor pregnancy outcome	Yes	84	21.3
	No	311	78.7
	Total	395	100.0

Complication during current pregnancy	Yes	29	7.3
	No	366	92.7
	Total	395	100.0

Duration of pregnancy	First TM	115	29.1
	Second TM	139	35.2
	Third TM	141	35.7
	Total	395	100.0

**Table 3:** Psychiatry, social support, and substance use of pregnant women attending ANC at health institutions of Faafan zone, December 2015 (*n* = 395).

Variable	Categories	Frequencies	Percent (%)
Previous depression history	Yes	41	10.4
	No	354	89.6
	Total	395	100.0

Social support	Poor	51	12.9
	Moderate	146	37.0
	Strong	198	50.1
	Total	395	100.0

Family history of depression	Yes	19	4.8
	No	376	95.2
	Total	395	100.0

Khat use	Yes	44	11.1
	No	351	88.9
	Total	395	100.0

Tobacco use	Yes	17	4.3
	No	378	95.7
	Total	395	100.0

Alcohol use	Yes	2	.5
	No	393	99.5
	Total	395	100.0

Cannabis use	Yes	1	.3
	No	394	99.7
	Total	395	100.0

**Table 4:** Predictors of antenatal depression among pregnant women attending ANC at health institutions of Faafan zone, December 2015 (*n* = 395).

Variable		Antenatal depression		AOR (95% CI)
		Yes = 96	No = 299	
Marital status	Married and living together	64 (66.7%)	257 (86.0%)	1.0
	Married but not living together	19 (19.8%)	31 (10.4%)	2.91 (1.36, 6.23)
	Divorced and single	11 (11.4%)	9 (%)	3.21 (0.93, 11.02)
	Widowed	2 (2.1%)	2 (.7%)	1.99 (0.18, 3.46)

Educational status	Illiterate	54 (56.2%)	154 (51.5%)	2.49 (1.09, 5.71)
	Primary	30 (31.2%)	82 (27.4%)	1.80 (0.75, 4.37)
	Secondary and above	12 (12.5%)	63 (21.1%)	1.0

Chronic medical illness	Yes	21 (21.9%)	88 (29.4%)	2.21 (1.09, 4.45)
	No	75 (78.1%)	211 (70.6%)	1.0

Number of pregnancy	First	26 (27.1%)	51 (17.1%)	8.89 (2.09, 12.78)
	Second	30 (31.2%)	90 (30.1%)	7.32 (1.81, 11.97)
	Third	20 (20.8%)	70 (23.4%)	5.77 (1.38, 9.41)
	Fourth	6 (6.2%)	26 (8.7%)	4.73 (0.89, 7.49)
	Fifth and above	14 (14.6%)	62 (20.7%)	1.0

Previous depression history	Yes	31 (32.3%)	10 (3.3%)	6.02 (2.31, 10.01)
	No	161 (67.7%)	295 (96.7%)	1.0

Social support	Poor	28 (29.2%)	23 (7.7%)	3.34 (1.50, 7.43)
	Moderate	35 (36.5%)	111 (37.1%)	.990
	Strong	33 (34.4%)	166 (55.2%)	1.0

## Data Availability

Data is available on the hand of the authors.

## Abbreviations

ANC: Antenatal care
AD: Antenatal depression
AOR: Adjusted odds ratio
BDI: Becky depression inventory
CI: Confidence interval
EDHS: Ethiopian Demographic and Health Survey
EPDS: Edinburgh Postnatal Depression Scale
OR: Odds ratio
OSS-3: Oslo-3 Social Support Scale
SD: Standard deviation
SPSS: Software Package for Social Science
SSA: Sub-Saharan Africa.
